# A59 EXPLORING THE ROLE OF ARID1A IN COLONIC HOMEOSTASIS AND REGENERATION

**DOI:** 10.1093/jcag/gwad061.059

**Published:** 2024-02-14

**Authors:** D Lei, A Loe, T Kim

**Affiliations:** University of Toronto, Toronto, ON, Canada; Developmental and Stem Cell Biology, SickKids Research Institute, Toronto, ON, Canada; University of Toronto, Toronto, ON, Canada

## Abstract

**Background:**

Recent advances in cancer genome analysis have revealed frequently mutated epigenetic regulators as a novel feature in cancer development. Within this context, the *Arid1a* (AT-rich interactive domain-containing protein 1A) gene, a subunit of the BAF chromatin remodeling complex, has emerged as a frequently mutated gene in many cancers. Additionally, repeated colonic injury caused by diseases such as inflammatory bowel disease (IBD), is also a major risk factor leading to colorectal cancer. Therefore, understanding the connection between colonic epigenetic regulation and its role in response to injury is particularly important for understanding the pathobiology of colorectal cancer.

**Aims:**

Aim 1: Exploring the role of *Arid1a* during colonic homeostasis.

Mouse model with *Arid1a* conditionally knocked out in the colon will be analysed at different time points to test whether *Arid1a* is required during colonic homeostasis.

Aim 2: Determining the role of *Arid1a* during colonic injury and regeneration.

Mice will be exposed to a known colitis model to induce colonic injury. *Arid1a* will be deleted after injury to find out its role during regeneration and recovery from the injury.

**Methods:**

To examine the effect of *Arid1a* loss on colonic homeostasis, mice between 6-10 weeks old were injected with 2mg/20g tamoxifen for three days to induce *Arid1a* knockout using the *VilCreERT2; Arid1afl/fl* mousseline. Mice were then euthanised using CO2 chamber and analysed 10 days, 3 weeks, and 8 weeks post *Arid1a* deletion. Histological analysis was performed to examine morphological changes in the colon. To examine the effect of *Arid1a* on injury caused by colitis, mice were treated with dextran sulfate sodium (DSS) to cause colitis-like injury. Then,

*Arid1a* is deleted by injecting 2mg/20g tamoxifen. Changes in histology and specific cell types will be examined both in the short- and long-term.

**Results:**

To test whether *Arid1a* loss alone causes morphological changes in the colon, I compared the crypt lengths between *VilCreERT2; Arid1afl/fl* mutant mice with their littermate controls and saw that there was no difference in both morphology and length of the crypts. My results show that

*Arid1a* loss in the colon after injury resulted in impaired regeneration characterized by the prolonged loss of goblet cells and cryptal structure in the distal colon. In the long term, *Arid1a* loss before or after DSS treatment both lead to tumor formation, suggesting that *Arid1a* is required for colonic regeneration and repair while suppressing tumorigenesis.

**Conclusions:**

Based on my results, I conclude that *Arid1a* deletion alone does not cause significant morphological changes in the colon, but this loss affects colonic regeneration and recovery compared to colons with *Arid1a* intact. In the long term, the dysregulated colonic regeneration may lead to tumorigenesis.

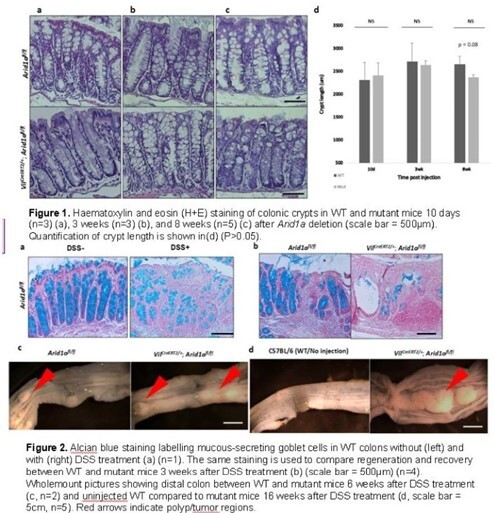

**Funding Agencies:**

University of Toronto Department of Molecular Genetics

